# Transcranial Direct Current Stimulation Over Prefrontal Areas Improves Psychomotor Inhibition State in Patients With Traumatic Brain Injury: A Pilot Study

**DOI:** 10.3389/fnins.2020.00386

**Published:** 2020-05-20

**Authors:** Xu Zhang, Baohu Liu, Nan Li, Yuanyuan Li, Jun Hou, Guoping Duan, Dongyu Wu

**Affiliations:** ^1^Department of Rehabilitation, Wangjing Hospital of China Academy of Chinese Medical Sciences, Beijing, China; ^2^Research Center of Clinical Epidemiology, Peking University Third Hospital, Beijing, China

**Keywords:** brain stimulation, transcranial direct current stimulation, traumatic brain injury, neurobehavioral disorder, non-linear dynamics, electroencephalogram

## Abstract

**Objectives:**

Many post-traumatic patients with minimally conscious state are complicated by psychomotor inhibition state (PIS), which impedes further rehabilitation. The treatment of PIS is not satisfactory. This pilot study aimed to investigate effects of anodal transcranial direct current stimulation (A-tDCS) on PIS in post-traumatic patients and examine the altered cortical activation after tDCS using non-linear electroencephalogram (EEG).

**Methods:**

The study included 10 patients with post-traumatic PIS. An A–B design was used. The patients received 4 weeks of sham tDCS during Phase A, and they received A-tDCS over the prefrontal area and left dorsolateral prefrontal cortex (DLPFC) for 4 weeks (40 sessions) during Phase B. Conventional treatments were administered throughout both phases. JFK Coma Recovery Scale-Revised (CRS-R), apathy evaluation scale (AES), and the EEG non-linear indices of approximate entropy (ApEn) and cross approximate entropy (C-ApEn) were measured before Phase A, before Phase B, and after Phase B.

**Results:**

After A-tDCS treatment, CRS-R and AES were improved significantly. ApEn and C-ApEn results showed that the local cortical connection of bilateral sensorimotor areas with their peripheral areas could be activated by affected painful stimuli, while bilateral cerebral hemispheres could be activated by the unaffected painful-stimuli condition. Linear regression analysis revealed that the affected sensorimotor cortex excitability and unaffected local and distant cortical networks connecting the sensorimotor area to the prefrontal area play a major role in AES improvement.

**Conclusion:**

A-tDCS over the prefrontal area and left DLPFC improves PIS. The recovery might be related to increased excitability in local and distant cortical networks connecting the sensorimotor area to the prefrontal area. Thus, tDCS may be an alternative treatment for post-traumatic PIS.

## Introduction

The disorders of consciousness (DoCs) are common in patients with severe traumatic brain injury (TBI). Such patients usually experience coma, unresponsive wakefulness state (UWS), and minimally conscious state (MCS). Many patients may remain UWS or MCS for a prolonged period even if they survive the coma (Leonardi et al., 2013). However, many MCS patients, who have given great hope to the doctors and their families, entered into a psychomotor inhibition state (PIS), which is manifested as apathy, lack of desire for active communication, no appetite for food, and lack of motive for purposeful activities.

Neurobehavioral disorders (NDs) are classified into four types based on the subacute and chronic stages of severe TBI. The disorders of excessive behavior (for example, disinhibition, irritability, and aggression) and deficient behavior, such as apathy and depression, involve distinct brain regions (orbitofrontal for disorders of excess) or systems (orbitofrontal–amygdala) and imbalance of neurotransmitters (dopaminergic for attention) or hormones (Levin, 2016). PIS is one of the behavioral deficits in ND. Epidemiological investigation showed that the incidence of mental–behavioral disorders within 1 year after TBI was 49% (Zasler et al., 2013). The results of a prospective multicenter study showed excessive behavioral disorders in less than 3 months of trauma, while the incidence of deficient behavior disorder is higher in the chronic course (Nygren DeBoussard et al., 2017).

Presently, the pathogenesis of psychomotor inhibition is not quite clear. Some studies have shown that an impaired prefrontal–amygdala pathway may be a putative cause of inhibitive NDs (Fischer et al., 2014). Other studies have shown that different types of apathy (emotional, cognitive, and behavioral) have diverse pathogenesis. Among these, emotional type is related to the frontal marginal area (orbitofrontal cortex) and the ventral striatum, the cognitive type is related to the frontal lobe [dorsal prefrontal cortex (PFC)] and the dorsal caudate nucleus, and the behavioral type is associated with the bilateral prefrontal–basal ganglia circuit damage (Levy, 2012). In addition, the dorsolateral prefrontal cortex (DLPFC) damage is also associated with apathy and lack of will. Occasionally, apathy related to the middle of the frontal lobe damage is often mistaken for severe depression (Zasler et al., 2013).

The current clinical interventions for deficient behavior include environmental enrichment therapy and antidepressant drugs (such as amantadine). These treatments have limited efficacy but adverse drug reactions (Neurobehavioral Guidelines Working Group, Warden et al., 2006). Therefore, finding adequate interventions is an urgent clinical requisite.

Non-invasive brain stimulation methods are utilized for the treatment of mental disorders and NDs after brain trauma (Dhaliwal et al., 2015). Transcranial direct current stimulation (tDCS) regulates cortical excitability with a weak current of 1–2 mA, and anodal tDCS (A-tDCS) increases the cortical excitability (Merzagora et al., 2010). We also used tDCS to study DoC, aphasia, dysphagia, and limb dysfunction for optimal clinical efficacy. A-tDCS over the left posterior perisylvian region improves picture naming in new-onset aphasic patients (Wu et al., 2015) and also improves the language performance of non-fluent variant primary progressive aphasia (Wang et al., 2013), which in turn, upregulates the excitability of a language network. Moreover, A-tDCS could improve swallowing apraxia by increasing the excitability of the swallowing cortex (Yuan et al., 2017). Cathodal tDCS could reduce muscle tone by inhibiting the hyperactivation of ipsilesional primary sensorimotor cortex (Wu et al., 2013).

Electroencephalogram (EEG) is a commonly used bedside electrophysiological assessment technique for the dynamic monitoring of brain function in the clinic. Non-linear science has become a new hot spot in the study of brain function using the principles and methods of non-linear dynamics such as approximate entropy (ApEn) and cross-ApEn (C-ApEn). Our previous study shows that non-linear dynamic analysis (NDA) characterizes the changes in the brain function for unconscious state and might be valuable in predicting the prognosis of unconscious subjects (Wu et al., 2011a). C-ApEn measures the interconnection of the residual cortical functional islands with associative cortices in the DoC patients. C-ApEn in UWS is significantly lower than that in MCS (Wu et al., 2011b). In addition, NDA could reflect the cortical activation during language (Wang et al., 2013; Wu et al., 2015) and swallowing (Yuan et al., 2017) tasks. According to previous research, we put forward the hypothesis that A-tDCS improves the psychomotor inhibition in post-traumatic patients recovering from DoC and that the underlying therapeutic mechanism might be related to the improvement of frontal cortical excitability and increase in the interconnection in the functional cortex.

The present study aimed to test the hypothesis. A-tDCS was applied over the prefrontal and the left DLPFCs in 10 PIS patients who recovered from DoC. ApEn and C-ApEn were measured for all subjects to investigate the change of cortical activation after tDCS.

## Materials and Methods

### Subjects

The study was performed in the Department of Rehabilitation, Wangjing Hospital of China Academy of Chinese Medicine Sciences. The cohort of PIS subjects with severe brain trauma consisted of seven males and three females, aged 16–83 years. The duration of injury ranged from 94 to 294 (average, 128.8) days. All subjects were right-handed according to the Edinburgh Handedness Inventory by inquiring their guardians or parents. Informed written consent was obtained from their guardians or parents. The hospital ethics committee approved this study.

Inclusion criteria were as follows: (1) all subjects were TBI; (2) onset of brain injury was >3 months before participation in the study; (3) all subjects recovered from UWS; (4) all subjects were diagnosed as MCS according to the JFK Coma Recovery Scale-Revised (CRS-R); (5) all subjects manifested deficient behavior and apathy; (6) no antidepressant drugs were used.

Exclusion criteria were as follows: (1) unstable vital signs; (2) epilepsy; (3) regional skin injury under the tDCS electrode; (4) subject having severe spasticity causing an EMG artifact.

### Design and Procedures

This study used an A–B design. In Phase A, sham tDCS was administered for 4 weeks; in Phase B, active tDCS was executed for 4 weeks. Conventional treatments were administered throughout both phases.

According to our preexperiment, the mean difference of AES was 13. We could not get the standard deviation (SD) of AES in post-traumatic PIS patients directly from the existing literatures. According to Marin’s result of AES (Marin et al., 1991), the SDs of AES-C in left-hemisphere stroke and Alzheimer’s disease were all 11.7, which was higher than that in right-hemisphere stroke and major depression. Therefore, the higher SD was chosen to calculate the sample size. Sample size was determined on the following parameters: α = 0.05, 1−β = 0.9, AES means of difference was 13, and SD was 11.7. Then the total sample size was 9, and the actual power is 0.9176 (according to G^∗^power, version 3.1.9.4). Our study included 10 cases.

### Interventions

Direct current was delivered by a portable battery-driven device (IS200, Chengdu, China). A constant current of 2.0 mA (0.056 mA/cm^2^) was applied using a saline-soaked pair of surface sponge electrodes (5 cm × 7 cm) for 20 min twice daily, 5 days/week for 4 weeks (40 sessions).

**FIGURE 1:**
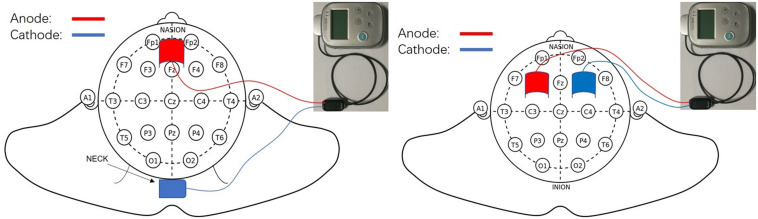
Transcranial direct current stimulation electrode position.

The daily tDCS treatment included A-tDCS over the prefrontal area in the morning and left DLPFC in the afternoon (two sessions). Using the international 10–20 system, the prefrontal area was located at 3.5 cm above FPz (the electrode was placed vertically with a lower edge at the FPz level), according to our preliminary experimental results. The left DLPFC was identified by the F3 electrode position (O’Neil-Pirozzi et al., 2017). The former cathode electrode was placed over the neck, and the latter was placed over F4 (Figure 1).

Stimulation parameters of sham tDCS were similar to our published study (Wu et al., 2013). The electrodes were placed in the same position, but sham tDCS lasted only 30 s. This procedure blinded all subjects and their guardians to the respective stimulation conditions.

Immediately after tDCS, the conventional treatments were applied for 30 min two times daily. Conventional treatments included (1) multimodal sensory stimulation and auditory stimulation; (2) bedside conventional physical therapy including maintaining good limb position, chronic stretching, and physical modalities and techniques; and (3) environmental enrichment therapy (sensory enrichment therapy, auditory enrichment therapy, and visual enrichment therapy).

### Blinding

Phases A and B were assigned 1 and 2, respectively. The assigned number (1 or 2) was inputted into the stimulator device by a single investigator. She did not participate in other parts of the study. The device automatically generated active or sham tDCS according to the parity of the assigned number. All other investigators, subjects, their guardians, and outcome assessors remained blinded to the details of the treatment until the completion of the final statistical analyses.

### Clinical Assessment

The CRS-R (Kalmar and Giacino, 2005), developed by the JFK Medical Center, was used to assess the patients’ consciousness. The comprehensive assessment was made from six aspects: auditory function, oromotor/verbal function, motor function, visual function, communication, and arousal, with a total score of 23. The apathy evaluation scale (AES) (Marin, 1996) was used to evaluate the daily behavior and communication initiative of the subjects. The AES, based on interviews, assessed the patients’ cognitive, behavioral, emotional, and routine arrangements in the past 4 weeks based on the oral and non-oral information from patients and their families. The AES consists of 18 items on a 4−point Likert scale (range: 18–72). The items were scored from 1 to 4 (items 6, 10, and 11 were three inverted items). A higher score indicated a greater apathy.

The two scales were evaluated before Phase A, before Phase B, and after Phase B. All the assessments were conducted by two doctors who had more than 5 years of experience in the evaluation and treatment of DoC. In addition, subjects who were discharged from the hospital after 6 months were investigated via telephone calls to assess the functional prognosis with the Glasgow outcome scale (GOS).

### EEG Recording

Electroencephalogram signals were recorded with a wireless digital EEG system (ZN16E, Chengdu, China). The main parameters were as follows: bandwidth 0.3–100 Hz, sample rate 500 Hz. In this study, 16 EEG electrodes were used according to the international 10–20 system The reference was earlobe electrodes. A hard drive was used to store data for further analysis. During the entire recording process, the subjects were awake and lay comfortably in a quiet ward.

Electroencephalogram was recorded under two conditions: eyes closed (5 min) and painful stimuli (first the affected side and then the unaffected side). While EEG was recorded, Quchi (LI11), Neiguan (PC6), Hegu (LI4), Waiguan (SJ5), Zusanli (ST36), Sanyinjiao (SP6), Taichong (LR3), and Yongquan (KI1) acupuncture points were electrically stimulated by using a Han acupoint nerve stimulator (HANS). HANS ensured that every acupoint would get the same current intensity. Evidence from functional MRI (fMRI) supports the view that stimulation of acupuncture points ST36, SP6, LR3, KI1, PC6, TE5, DU26, LI4, and LI11 could activate primary and secondary somatosensory areas as well as other subcortical areas such as the insula, thalamus cerebellum, DLPFC, and parahippocampal gyrus (Zhang et al., 2004; Yan et al., 2005; Bai et al., 2010; Cho et al., 2010; Chen et al., 2014; Chae et al., 2015). Therefore, pain stimulation caused by HANS was used to reach the maximal effect of cortical activation.

The methods of avoiding the EMG artifact during EEG recording was similar to the previous studies (Wu et al., 2011a, b).

An artifact-free epoch was chosen off-line by an experienced physician. To exclude the electrical noise, a 50-Hz notch filter was used.

Since the affected side of the brain injury for subjects was variable, we used the affected and unaffected sides accordingly. Thus, the electrodes were changed to FP_A_, FP_U_, F_A_, F_U_, C_A_, C_U_, P_A_, P_U_, O_A_, O_U_, AT_A_ (anterior temporal), AT_U_, MT_A_ (middle temporal), MT_U_, PT_A_ (posterior temporal), and PT_U_. For bilateral hemispheric injury, the affected side was assigned to the severe side of functional impairment.

### Non-linear Dynamics Analysis

Approximate entropy and C-ApEn were calculated to determine the cortical response to the PIS subjects. The program used in this study was similar to that use in our previous study (Wu et al., 2011a, b).

Approximate entropy assigns a non-negative number to a time series, with larger values corresponding to more complexity or irregularity in the data. Thus, increasing irregularity will cause higher complexity in the time series, that is, raised non-linear cell dynamics or interaction of cortical networks (increased ApEn).

Cross approximate entropy is very similar to ApEn in design and intent, differing only in that it compares sequences from one series with those of the second. While the single-channel ApEn measures the temporal complexity of the EEG, the two-channel C-ApEn reflects the spatial decorrelation of cortical potentials from two remote sites. Moreover, C-ApEn may be interpreted as a measure of the number of states independently accessible by the two cortical areas. Thus, an increase in C-ApEn during painful stimuli may indicate not only an increase in the number of independent microstates available for the two cortical areas but also increased intercortical communication or information flow.

The C_A_ and C_U_ with other EEG sites were calculated to determine the cortical response to the PIS subjects with painful stimuli (Hudetz, 2002). Local and distant C-ApEns were calculated in order to assess whether the changes in C-ApEn were associated with the impaired transmission of information over short or long distances. The local C-ApEn (around the C_A_ and C_U_) consisted of C_A_–F_A_, C_U_–F_U_, C_A_–MT_A_, C_U_–MT_U_, C_A_–P_A_, and C_U_–P_U_. The distant C-ApEn consisted of C_A_–FP_A_, C_U_–FP_U_, C_A_–O_A_, and C_U_–O_U_.

### Statistical Analysis

SPSS (version 22) was used for the analyses. A paired *t*-test was used to compare the changes in CRS-R and AES before and after the treatments and the changes in ApEn and C-ApEn difference between painful-stimuli and eyes-closed conditions before and after the treatment. A linear regression model was established using the forward method. The characteristics of ApEn, C-ApEn, and patient’s characteristics (age, gender, and duration) were considered as independent variables, while CSR-R and AES were taken as dependent variables to construct the regression model. Statistical significance was determined at *p* < 0.05.

## Results

The clinical characteristics of all subjects are listed in Table 1. None of the subjects withdrew from the study.

**TABLE 1:** Clinical characteristics of the patients.

ID	Age (y)	Duration (d)	Initial diagnosis	MRI/CT findings	GOS
1	21–25	112	MCS	Right frontal, left basal ganglia, midbrain hemorrhage	5
2	31–35	167	MCS	Diffused cerebral edema	4
3	36–40	97	MCS	Bilateral frontal hemorrhage	4
4	56–60	113	MCS	Left frontal, temporal, and occipital hemorrhage with midline shift	4
5	81–85	116	MCS	Bilateral frontal hemorrhage	4
6	16–20	104	MCS	Diffused cerebral edema	5
7	51–55	96	MCS	Left frontal hemorrhage, left frontoparietal white matter hyperintensity	5
8	66–70	294	MCS	Left frontotemporal, right frontal hemorrhage	4
9	81–85	94	MCS	Bilateral frontal hemorrhage	4
10	81–85	95	MCS	Left frontal and temporal hemorrhage, left sub-dural hematoma with midline shift	4
					

### Clinical Assessment

**FIGURE 2:**
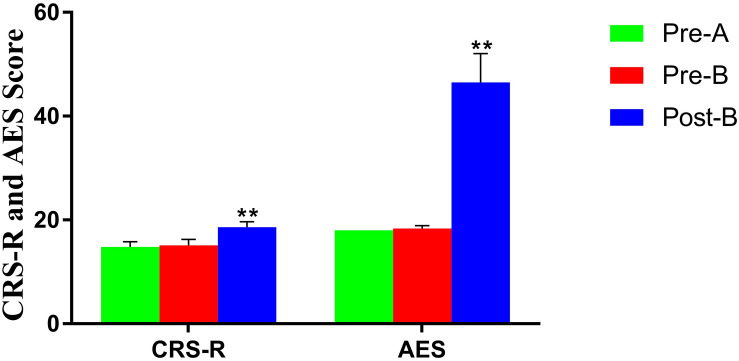
Altered CRS-R and AES in patients before and after treatments. **Significant *p*-value: *p* < 0.01. The results showed that CRS-R and AES were improved significantly after treatments.

No differences in CRS-R and AES were found between Pre-A and Pre-B. After Phase B, CRS-R, and AES were improved significantly (Table 2 and Figure 2).

**TABLE 2:** CRS-R and AES in patients before and after the treatments.

ID	CRS-R			AES		
	Pre-A	Pre-B	Post-B	Pre-A	Pre-B	Post-B
1	14	15	19	18	18	42
2	14	14	18	18	19	43
3	14	14	18	18	18	42
4	15	15	18	18	18	46
5	15	16	19	18	19	52
6	16	17	19	18	19	56
7	17	17	21	18	19	54
8	14	14	17	18	18	41
9	15	15	19	18	18	47
10	14	14	18	18	18	42

*CRS-R, the JFK coma recovery scale-revised; AES, the apathy evaluation scale; Pre-A, before Phase A; Pre-B, before Phase B; Post-B, after Phase B.*

### ApEn Analysis

**FIGURE 3:**
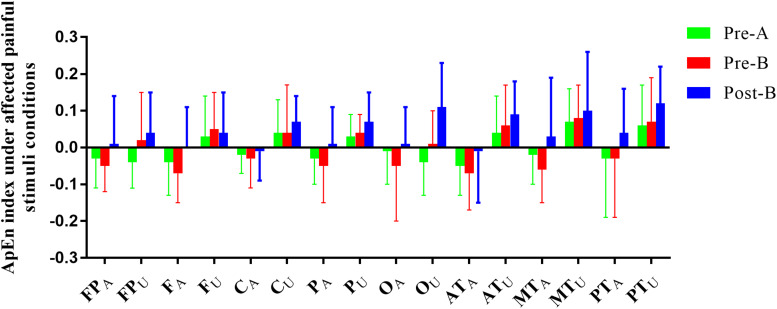
ApEn index under the affected painful-stimuli condition.

**FIGURE 4:**
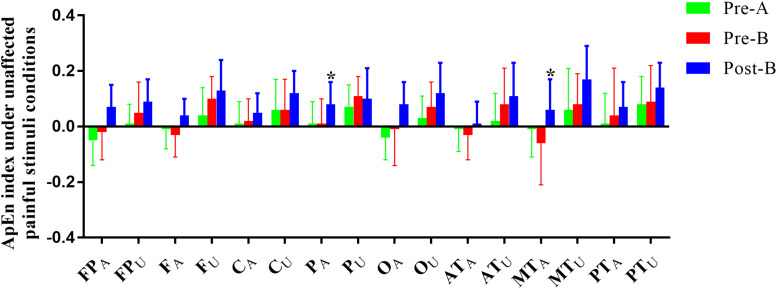
ApEn index under the unaffected painful-stimuli condition. *p < 0.05 was considered statistically significant.

Electroencephalogram ApEn difference between painful-stimuli and eyes-closed conditions before and after the treatments are listed in Table 3 (Figures 3, 4). There was no difference in either of the electrodes between Pre-A and Pre-B under bilateral painful stimuli. After Phase B, no significant difference was observed in either of the electrodes under affected painful stimuli; however, the ApEn indices were significantly higher in P_A_ (affected parietal) and MT_A_ (affected middle temporal) under unaffected painful stimuli.

**TABLE 3:** Changes in the ApEn difference between painful stimuli conditions and eyes-closed condition before and after the treatments.

Montage	Under affected painful stimuli conditions						Under unaffected painful stimuli conditions					
	Pre-A	Pre-B	*p*	Pre-B	Post-B	*p*	Pre-A	Pre-B	*p*	Pre-B	Post-B	*p*
FP_A_	−0.03 ± 0.08	−0.05 ± 0.07	0.824	−0.05 ± 0.07	0.01 ± 0.13	0.229	−0.05 ± 0.09	−0.02 ± 0.10	0.559	−0.02 ± 0.10	0.07 ± 0.08	0.096
FP_U_	−0.04 ± 0.07	0.02 ± 0.13	0.272	0.02 ± 0.13	0.04 ± 0.11	0.663	0.01 ± 0.07	0.05 ± 0.11	0.197	0.05 ± 0.11	0.09 ± 0.08	0.477
F_A_	−0.04 ± 0.09	−0.07 ± 0.08	0.182	−0.07 ± 0.08	−0.00 ± 0.11	0.198	−0.01 ± 0.07	−0.03 ± 0.08	0.347	−0.03 ± 0.08	0.04 ± 0.06	0.129
F_U_	0.03 ± 0.11	0.05 ± 0.10	0.277	0.05 ± 0.10	0.04 ± 0.11	0.906	0.04 ± 0.10	0.10 ± 0.08	0.782	0.10 ± 0.08	0.13 ± 0.11	0.268
C_A_	−0.02 ± 0.05	−0.03 ± 0.08	0.128	−0.03 ± 0.08	−0.01 ± 0.08	0.657	0.01 ± 0.08	0.02 ± 0.08	0.512	0.02 ± 0.08	0.05 ± 0.07	0.328
C_U_	0.04 ± 0.09	0.04 ± 0.13	0.695	0.04 ± 0.13	0.07 ± 0.07	0.645	0.06 ± 0.11	0.06 ± 0.11	0.782	0.06 ± 0.11	0.12 ± 0.08	0.273
P_A_	−0.03 ± 0.07	−0.05 ± 0.10	0.295	−0.05 ± 0.10	0.01 ± 0.10	0.297	0.01 ± 0.08	0.01 ± 0.09	0.769	0.01 ± 0.09	0.08 ± 0.08	**0.013**
P_U_	0.03 ± 0.06	0.04 ± 0.05	0.826	0.04 ± 0.05	0.07 ± 0.08	0.376	0.07 ± 0.08	0.11 ± 0.07	0.603	0.11 ± 0.07	0.10 ± 0.11	0.787
O_A_	−0.01 ± 0.09	−0.05 ± 0.15	0.282	−0.05 ± 0.15	0.01 ± 0.10	0.189	−0.04 ± 0.08	−0.01 ± 0.13	0.351	−0.01 ± 0.13	0.08 ± 0.08	0.099
O_U_	−0.04 ± 0.09	0.01 ± 0.09	0.545	0.01 ± 0.09	0.11 ± 0.12	0.157	0.03 ± 0.08	0.07 ± 0.09	0.782	0.07 ± 0.09	0.12 ± 0.11	0.398
AT_A_	−0.05 ± 0.08	−0.07 ± 0.10	0.174	−0.07 ± 0.10	−0.01 ± 0.14	0.324	−0.01 ± 0.08	−0.03 ± 0.09	0.729	−0.03 ± 0.09	0.01 ± 0.08	0.228
AT_U_	0.04 ± 0.10	0.06 ± 0.11	0.159	0.06 ± 0.11	0.09 ± 0.09	0.539	0.02 ± 0.10	0.08 ± 0.13	0.855	0.08 ± 0.13	0.11 ± 0.12	0.722
MT_A_	−0.02 ± 0.08	−0.06 ± 0.09	0.179	−0.06 ± 0.09	0.03 ± 0.16	0.105	−0.01 ± 0.10	−0.06 ± 0.15	0.169	−0.06 ± 0.15	0.06 ± 0.11	**0.033**
MT_U_	0.07 ± 0.09	0.08 ± 0.09	0.272	0.08 ± 0.09	0.10 ± 0.16	0.758	0.06 ± 0.15	0.08 ± 0.11	0.549	0.08 ± 0.11	0.17 ± 0.12	0.125
PT_A_	−0.03 ± 0.16	−0.03 ± 0.16	0.365	−0.03 ± 0.16	0.04 ± 0.12	0.265	0.01 ± 0.11	0.04 ± 0.17	0.434	0.04 ± 0.17	0.07 ± 0.09	0.566
PT_U_	0.06 ± 0.11	0.07 ± 0.12	0.760	0.07 ± 0.12	0.12 ± 0.10	0.425	0.08 ± 0.10	0.09 ± 0.13	0.729	0.09 ± 0.13	0.14 ± 0.09	0.350

*Values are mean ± standard deviation. Bold significant *p*-value: *p* < 0.05 was considered statistically significant.*

### C-ApEn Analysis

**FIGURE 5:**
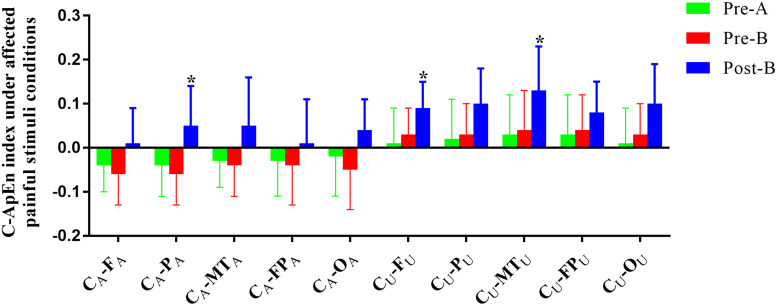
C-ApEn index under the affected painful-stimuli condition. *p < 0.05 was considered statistically significant.

**FIGURE 6:**
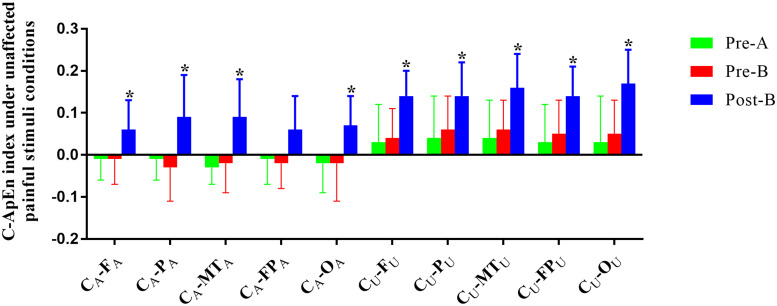
C-ApEn index under the unaffected painful-stimuli condition. *p < 0.05 was considered statistically significant.

The EEG C-ApEn difference between painful-stimuli and eyes-closed conditions before and after the treatments is listed in Table 4 (Figures 5, 6). There was no difference in either of the electrodes between Pre-A and Pre-B under bilateral painful stimuli. After Phase B, the local C-ApEn indices of C_A_–P_A_, C_U_–F_U_, and C_U_–MT_U_ were significantly higher under the affected painful-stimuli condition, while almost all local and distant C-ApEn indices were significantly higher under the unaffected painful-stimuli condition.

**TABLE 4:** Changes in the C-ApEn difference between painful stimuli conditions and eyes-closed condition before and after the treatments.

Montage	Under affected painful stimuli conditions						Under unaffected painful stimuli conditions					
	Pre-A	Pre-B	*p*	Pre-B	Post-B	*p*	Pre-A	Pre-B	*p*	Pre-B	Post-B	*p*
C_A_-F_A_	−0.04 ± 0.06	−0.06 ± 0.07	0.512	−0.06 ± 0.07	0.01 ± 0.08	0.128	−0.01 ± 0.05	−0.01 ± 0.06	0.214	−0.01 ± 0.06	0.06 ± 0.07	**0.048**
C_A_-P_A_	−0.04 ± 0.07	−0.06 ± 0.07	0.761	−0.06 ± 0.07	0.05 ± 0.09	**0.023**	−0.01 ± 0.05	−0.03 ± 0.08	0.654	−0.03 ± 0.08	0.09 ± 0.10	**0.015**
C_A_-MT_A_	−0.03 ± 0.06	−0.04 ± 0.07	0.681	−0.04 ± 0.07	0.05 ± 0.11	0.081	−0.03 ± 0.04	−0.02 ± 0.07	0.594	−0.02 ± 0.07	0.09 ± 0.09	**0.028**
C_A_-FP_A_	−0.03 ± 0.08	−0.04 ± 0.09	0.824	−0.04 ± 0.09	0.01 ± 0.10	0.272	−0.01 ± 0.06	−0.02 ± 0.06	0.813	−0.02 ± 0.06	0.06 ± 0.08	0.062
C_A_-O_A_	−0.02 ± 0.09	−0.05 ± 0.09	0.729	−0.05 ± 0.09	0.04 ± 0.07	0.081	−0.02 ± 0.07	−0.02 ± 0.09	0.782	−0.02 ± 0.09	0.07 ± 0.07	**0.043**
C_U_-F_U_	0.01 ± 0.08	0.03 ± 0.06	0.471	0.03 ± 0.06	0.09 ± 0.06	**0.006**	0.03 ± 0.09	0.04 ± 0.07	0.437	0.04 ± 0.07	0.14 ± 0.06	**0.000**
C_U_-P_U_	0.02 ± 0.09	0.03 ± 0.07	0.247	0.03 ± 0.07	0.10 ± 0.08	0.060	0.04 ± 0.10	0.06 ± 0.08	0.849	0.06 ± 0.08	0.14 ± 0.08	**0.001**
C_U_-MT_U_	0.03 ± 0.09	0.04 ± 0.09	0.622	0.04 ± 0.09	0.13 ± 0.10	**0.011**	0.04 ± 0.09	0.06 ± 0.07	0.834	0.06 ± 0.07	0.16 ± 0.08	**0.001**
C_U_-FP_U_	0.03 ± 0.09	0.04 ± 0.08	0.665	0.04 ± 0.08	0.08 ± 0.07	0.305	0.03 ± 0.09	0.05 ± 0.08	0.225	0.05 ± 0.08	0.14 ± 0.07	**0.007**
C_U_-O_U_	0.01 ± 0.08	0.03 ± 0.07	0.842	0.03 ± 0.07	0.10 ± 0.09	0.069	0.03 ± 0.11	0.05 ± 0.08	0.645	0.05 ± 0.08	0.17 ± 0.08	**0.004**

*Values are mean ± standard deviation. Bold significant *p*-value: *p* < 0.05 was considered statistically significant.*

### Linear Regression Analysis

Tables 5, 6 list the results of relevant factors of CRS-R and AES improvement. Linear regression analysis showed that F_A_, C_U_, C_A_–P_A_, and C_A_–F_A_ were associated with the improvement in CRS-R under the affected painful-stimuli condition (Table 5). In addition, gender is another factor related to the improvement of CRS-R. However, the improved AES is related to C_A_ and C_U_–F_U_ under the affected painful-stimuli condition and C_U_–FP_U_ under the unaffected painful-stimuli condition (Table 6).

**TABLE 5:** Relevant factors of CRS-R improvement.

Model	Unstandardized coefficient		Standardized coefficienth	*t*	*p*
	B	Standard error	b		
Constant	4.16	0.03		148.71	**<0.001**
Improvement in C_A_-P_A_ (APS)	−7.34	0.07	−1.22	−103.63	**<0.001**
Improvement in C_A_-F_A_ (APS)	4.91	0.08	0.85	60.02	**<0.001**
Improvement in F_A_ (APS)	−2.11	0.06	−0.42	−33.62	**<0.001**
Improvement in C_U_ (APS)	−0.41	0.05	−0.08	−7.66	**0.005**
Gender	−0.06	0.02	−0.04	−3.47	**0.040**

*APS, under affected painful stimuli conditions. Bold significant *p*-value: *p* < 0.05 was considered statistically significant.*

**TABLE 6:** Relevant factors of AES improvement.

Model	Unstandardized coefficient		Standardized coefficient	*t*	*p*
	B	Standard error	b		
Constant	31.03	1.00		30.91	**<0.001**
Improvement of C_A_-FP_A_ (UPS)	−70.43	7.85	−0.90	−8.97	**<0.001**
Improvement of C_U_-F_U_ (APS)	58.16	10.61	0.55	5.48	**0.003**
Improvement of C_A_ (APS)	12.80	4.01	0.31	3.19	**0.024**

*APS, under affected painful stimuli conditions. UPS, under unaffected painful stimuli conditions. Bold significant *p* value: *p* < 0.05 was considered statistically significant.*

## Discussion

The results confirmed our hypotheses. First, A-tDCS over the prefrontal area and left DLPFC improves the psychomotor inhibition in post-traumatic patients recovering from DoC. After A-tDCS, both CRS-R and AES were improved significantly. Second, after A-tDCS, the ApEn indices were significantly higher in P_A_ and MT_A_ under the unaffected painful-stimuli condition. Interestingly, the C-ApEn indices were significantly higher in the local cortical network (C_A_–P_A_, C_U_–F_U_, and C_U_–MT_U_) under the affected painful-stimuli condition and in almost all local and distant cortical networks under the unaffected painful-stimuli condition. Furthermore, frontal excitability (F_A_) and interconnection of frontal with other areas (C_A_–F_A_, C_U_–F_U_, and C_U_–FP_U_) played a key role in the improvement in the relevant factors of CRS-R and AES. Therefore, the improvement in the CRS-R and AES could be interpreted as an increase in both local cortical excitability (affected sensorimotor and its peripheral cortices) and interconnection between the sensorimotor area and frontal area.

### Effect of tDCS Treatment

Clinically, patients with DoC after severe TBI should be under intensive focus. After the coma phase, patients can transit to UWS, MCS, or E-MCS and recover a full consciousness (Portaccio et al., 2018). However, many patients would remain UWS and MCS for a prolonged period and, finally, give up rehabilitation treatment. Some patients in MCS or E-MCS recovered some motor and communication functions, while many manifested PIS. PIS is the most prominent manifestation in this period that would impede the recovery progress of consciousness. A retrospective cohort study compared the long-term outcomes of 12 patients in the UWS and 39 patients in the MCS up to 5 years post-injury. Thirteen emerged from MCS to conscious state, while none of the patients in the UWS improved (Luaute et al., 2010). Moreover, 26 patients remained MCS for a long time. Among them, the actual number of patients who might be complicated by PIS was unknown. In the present study, 4-week conventional treatments still did not obtain a satisfactory effect.

Although the CRS-R score increased significantly after A-tDCS treatment, the result still could not adequately reflect the improved clinical manifestation. These patients exhibited a wide range of cognitive, behavioral, and emotional improvements in AES, which reflected the overall improvement, especially psychomotor inhibition. Moreover, the psychomotor inhibition and initiative of these patients improved significantly, which laid a foundation for further rehabilitation, such as speech–language therapy and cognitive rehabilitation. The GOS results also showed a favorable prognosis in all patients.

### Parameters of tDCS Used in This Study

#### *Status Quo* of tDCS Treatment of DoC

In recent years, tDCS has been applied for consciousness improvement in DoC patients. Naro et al. (2015) showed that a single session of A-tDCS (1 mA/25 cm^2^ × 10 min) over the orbitofrontal cortex in 25 patients with DoC (15 UWS and 10 MCS) could boost the cortical connectivity and excitability in MCS and unmask such excitability in some UWS patients. In addition, Bai et al. (2017) showed that a single session of A-tDCS (2 mA/25 cm^2^ × 20 min) over the left DLPFC in 18 patients with DoC (nine UWS and nine MCS) induces cortical excitability changes between UWS and MCS patients using TMS-EEG. Thibaut et al. (2014) demonstrated that a single session of A-tDCS (2 mA/35 cm^2^ × 20 min) over the left DLPFC in 55 patients with DoC (25 UWS and 30 MCS) might transiently improve the signs of consciousness in MCS following severe brain damage as measured by changes in CRS-R (1.6 ± 2.5) but no improvement in UWS.

Angelakis et al. (2014) using 10 sessions of A-tDCS (1 mA/25 cm^2^ × 20 min) over the left DLPFC in four patients with DoC (three UWS and one MCS) could improve the MCS− patient to MCS+ immediately after the treatment; however, no patient in UWS improved immediately after stimulation. Interestingly, the MCS patient who received a second round of tDCS (2 mA/25 cm^2^ × 20 min) at 3 months after the initial participation showed further improvement and emergence into consciousness after stimulation; but no change was detected in between the treatments.

In summary, these results were unsatisfactory. Some tDCS protocols targeting the left DLPFC in MCS have shown beneficial results. However, only preliminary data were reported with respect to the number of sessions and the amount and clinical profile of the patients awaiting treatment.

#### Why Were Multi-Sessions of tDCS Used?

Post-traumatic neural plasticity and functional reorganization were time-consuming processes. The review by Caeyenberghs et al. (2018) described that no studies had investigated the correlation between training duration and structural brain changes in the acquired brain injury and that training interventions culminate in robust effects if the training intervention is both intense and long term. The other reason was that our preliminary experimental results showed that multi-session tDCS treatment had a more favorable outcome than single-session tDCS for DoC patients. Therefore, 40 sessions of A-tDCS were used in this study, and the results reached a relatively satisfactory effect.

#### Why Were the Prefrontal Area and Left DLPFC Chosen?

At present, the PFC is believed to play a central role in the pathophysiology of psychopathy. Studies of brain structure and function in psychopathy have frequently identified abnormalities in the PFC. Subregions of the PFC mediate a variety of functions that contribute to behavioral control, social cognition, emotion, and value-based decision making. However, findings have not yet converged to yield a distinct correlation between specific subregions of the PFC and specific psychopathic traits (Kotchoubey et al., 2005). Another study suggested that the negative symptoms are similar to those seen with prefrontal lobe cortical dysfunction (Mattson et al., 1997). Moreover, the orbitofrontal cortex is involved in motivational behavior such as feeding and drinking, social behavior, and emotional behavior (Rolls, 2004). These evidences suggested that the function of PFC might be related to psychomotor inhibition.

In addition, neurophysiological deficits in the left DLPFC have been described in positron emission tomography studies of schizophrenia and depression and have been associated with the syndromes of psychomotor poverty and psychomotor retardation, respectively (Dolan et al., 1993). In this regard, an imbalance in favor of a higher activation in the right PFC was associated with impaired psychological responses such as reduced motivation and anxiety and high stress, while a high activation in the left PFC was associated with an improved psychological responses such as increased motivation and resilient behavior and high affect (Meyer et al., 2015). A systematic review suggested that a high left PFC activation is associated with a positive psychological response to exercise (Silveira et al., 2019). Some studies have shown that high-frequency repetitive transcranial magnetic stimulation (rTMS) increases the cortical excitability in the left DLPFC region to improve the negative symptoms of schizophrenia (Kamp et al., 2019) as well as a major depressive disorder (Corlier et al., 2019). Williams et al. (2014) have shown that direct stimulation of the left DLPFC might regulate the parietal attention network and affect the emotional expression of the limbic system. Thus, accumulating evidence suggested that cortical function in the left DLPFC region might be associated with psychomotor inhibition.

Therefore, in the present study, both the prefrontal area and left DLPFC were selected as A-tDCS targets.

### Evidence of Cortical Excitability and Interconnection

Approximate entropy results revealed that there was no significant difference in all the electrodes under the affected painful-stimuli condition after tDCS treatment, which might be associated with the severe impairment of the affected sensorimotor cortex. Under the unaffected painful-stimuli condition, the ApEn indices were significantly higher in P_A_ (affected parietal) and MT_A_ (affected middle temporal). The fMRI findings indicated that acupuncture stimulation resulted in brain activations in the primary sensory cortex, DLPFC, insula, postcentral gyrus, and temporal area (Chae et al., 2015; Li et al., 2018). This result indicated that the affected peripheral area of the primary sensory cortical excitability might be easily activated under the unaffected painful-stimuli condition after A-tDCS.

Cross approximate entropy results demonstrated that after A-tDCS, local C-ApEn indices of C_A_–P_A_, C_U_–F_U_, and C_U_–MT_U_ were significantly higher under the affected painful-stimuli condition, which indicated that local cortical connection of the bilateral sensorimotor areas with their peripheral areas could be activated by affected painful stimuli, while almost all the local and distant C-ApEn indices were significantly higher under the unaffected painful-stimuli condition, thereby indicating a widespread activation of the bilateral cerebral hemispheres. C-ApEn has a higher sensitivity than ApEn.

Linear regression analysis showed that F_A_, C_U_, C_A_–P_A_, and C_A_–F_A_ under the affected painful-stimuli condition were associated with the improvement in CRS-R and that the improvement in AES was related to C_A_ and C_U_–F_U_ under the affected painful-stimuli condition and C_U_–FP_U_ under the unaffected painful-stimuli condition. Mackeprang et al. (2002) suggested that the abnormality of sensorimotor gating (SG) is a crucial mechanism leading to mental disorders and that the neurological mechanism may be related to the dysfunction of cortico-striato-thalamo-cortical circuits localized in the midbrain and forebrain structures. The current results are in agreement with the SG theory that states that the frontal (F_A_ and F_U_), sensorimotor (C_A_, C_U_, and P_A_), and forebrain structures (FP_U_) might be the key to modulate the abnormality of SG. Therefore, affected sensorimotor cortex excitability (C_A_) and unaffected local and distant cortical networks connecting the sensorimotor area to the prefrontal area (C_U_-F_U_, C_U_-FP_U_) play a major role in AES improvement.

### Limitations

The small sample size was the main study limitation of this study. Also, the follow-up of this study was relatively simple and lacked objective assessment (such as EEG) and analysis of prognosis related to the time factor. In addition, we also do not know whether the patient would get further improvement with repeated tDCS treatment. Thus, a large sample size and standardized randomized controlled trial are needed for future studies. To assess lasting effects, a new study design should include more follow-up assessment methods, such as non-linear EEG combined with high-order cortical information processing (e.g., P300), which may improve assessment sensitivity and specificity.

## Conclusion

The application of A-tDCS stimulation to the prefrontal area and left DLPFC can significantly improve the symptoms ofpost-traumatic PIS patients in a short period. The recovery might be related to increased excitability in the local and distant cortical networks connecting the sensorimotor area to the prefrontal area. EEG NDA could provide the cortical excitability and the interconnection of the functional cortex. Thus, tDCS may be an add-on treatment for post-traumatic PIS.

## Data Availability Statement

The datasets generated for this study are available on request to the corresponding author.

## Ethics Statement

The studies involving human participants were reviewed and approved by The ethic committee of the Wangjing Hospital of CACMS. Written informed consent to participate in this study was provided by the participants’ legal guardian/next of kin.

## Author Contributions

XZ and BL made substantial contributions to the data analysis and drafting of the manuscript. NL made substantial contributions to the data statistics. YL, GD, and JH treated the patient and acquired the data. DW designed the study, supervised the initial drafting, and critically revised the manuscript. All authors read and approved the final manuscript.

## Conflict of Interest

The authors declare that the research was conducted in the absence of any commercial or financial relationships that could be construed as a potential conflict of interest.
